# A reduced genome decreases the host carrying capacity for foreign DNA

**DOI:** 10.1186/1475-2859-13-49

**Published:** 2014-04-01

**Authors:** Yuya Akeno, Bei-Wen Ying, Saburo Tsuru, Tetsuya Yomo

**Affiliations:** 1 Graduate School of Information Science and Technology, Osaka University, 1-5 Yamadaoka, Suita, Osaka 565-0871, Japan; 2 Graduate School of Life and Environmental Sciences, University of Tsukuba, Tsukuba, Ibaraki 305-8572, Japan; 3 Graduate School of Frontier Biosciences, Osaka University, Suita, Osaka 565-0871, Japan; 4 Exploratory Research for Advanced Technology (ERATO), Japan Science and Technology Agency (JST), Suita, Osaka 565-0871, Japan

**Keywords:** Reduced genome, Growth rate, Exogenous DNAs, Replication, Plasmid carrying capacity, Expression, *Escherichia coli*

## Abstract

**Background:**

Host-plasmid interactions have been discussed largely in terms of the influences of plasmids, whereas the contributions of variations in host genomes to host interactions with foreign DNA remain unclear. A strain with a so-called “clean genome” (*i.e.*, MDS42) of reduced genome size has recently been generated from the wild-type strain MG1655, a commonly used host strain. A quantitative evaluation of the influence of plasmid burdens in these two *Escherichia coli* strains can not only provide an understanding of how a reduced genome responds to foreign DNA but also offer insights into the proper application of these strains.

**Results:**

The decreases in growth caused by the cost of carrying foreign DNA were similar for the wild-type and clean-genome strains. A negative correlation between the growth rate and the total amount of exogenous DNA was observed in both strains, but a better theoretical fit with a higher statistical significance was found for the strain with the clean genome. Compared to the wild-type strain, the clean-genome strain exhibited a reduced carrying capacity for exogenous DNA, which was largely attributed to its ability to restrict the replication of foreign DNA. A tendency to allocate energy and resources toward gene expression, but not DNA replication, was observed in the strain with the clean genome.

**Conclusions:**

The possession of a clean genome constrained the plasmid copy number to a wild-type-equivalent load. The results indicate that the wild-type strain possesses a greater tolerance for foreign DNA, as in endosymbiosis, and that the use of strains with clean genomes will be favorable in the applications that require precise control and theoretical prediction.

## Background

Host-plasmid interactions have been intensively studied in both bacterial and yeast cells, with the aim of developing a quantitative understanding of how exogenous DNA, *i.e.*, plasmids, contribute to host growth [1-8]. Decreases in the growth rates of host cells that carry foreign DNA (*e.g.*, plasmids) have mainly been attributed to the additional costs associated with the expression of plasmid genes [9,10]. Because the size of the prokaryotic genome is considered to be constrained by bioenergetics, (gene expression across an entire genome is assumed to consume approximately 75% of the total energy produced by bacterial cells [11]), whether changes in the size of a host genome can improve a host’s capacity for DNA replication is an intriguing question.

Furthermore, evaluations of the contributions of host genomes to host-plasmid interactions are also relevant to biotechnological applications. A strain with a clean genome of reduced size (*i.e.*, MDS42) has recently been generated from the wild-type genome of strain MG1655 [12]. The MDS42 genome possesses significant advantages in terms of plasmid stability [13,14], protein production [15] and other applications in synthetic biology [16,17]. These advantages for biotechnological applications are largely attributed to genetic characteristics such as a low mutation rate and limited amount of recombination [13,14,17].

The latest genome-wide analyses have revealed that the reduced genome of MDS42 exhibits both a higher level of gene expression and a more fixed chromosomal periodicity [18] compared to its mother strain, MG1655 [19]. These novel properties of MDS42 largely result from the complete deletion of insertion sequences [12], which are considered to be nonessential for cellular life. Thus, MDS42 is believed to be an ideal host for plasmid production [20] because plasmids exhibit a greater degree of structural stability in MDS42 compared to MG1655 [12]. However, to date, the increase in the productivity of plasmids inserted into MDS42 has not been well studied [21].

In this study, we investigated whether a reduction in host genome size would benefit the replication of foreign DNA by comparing two closely related strains (*i.e.*, MG1655 and MDS42) of different genome sizes. Estimating the burden of foreign DNA (*i.e.*, plasmids) in both MG1655 and MDS42 allows us to determine how a reduction in genome size influences the carrying capacity for foreign DNA, and this information also provides further insight into the relationships among DNA replication, host growth and the cost distribution of cellular energetics. Any novel information concerning the nature of the host-plasmid interactions in strains with a clean genome (*e.g.*, MDS42) can be taken into consideration when choosing strains for various applications.

## Results and discussion

### The growth cost of foreign DNA is similar for a wild-type or a clean genome

**Figure 1:**
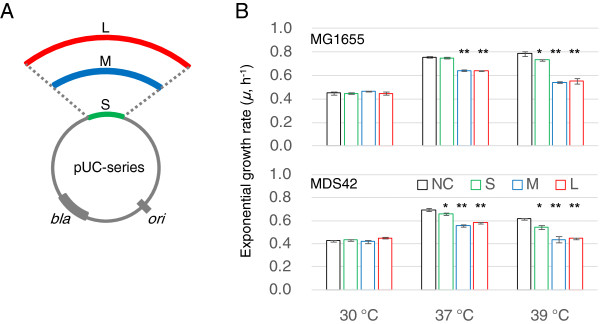
**Contributions of plasmids to cell growth. A**. Schematic drawing of the plasmids. All plasmids (pUC-S, pUC-M and pUC-L) were derived from pUC19. The lengths of S, M and L represent the plasmid sizes of 2,334, 6,426 and 9,573 bp, respectively. The site of initiation for replication and the ampicillin resistance gene are indicated as *ori* and *bla*, respectively. **B**. Cell growth rates. The exponential growth rates of *E. coli* strains MG1655 (upper panel) and MDS42 (lower panel) in a minimal medium are shown for different temperatures. The green, blue and red colors indicate the cells bearing the plasmids S, M and L, respectively. The strains that did not contain plasmids, which were used as controls (*i.e.*, native growth fitness), are shown in black. The bars represent the standard errors of triplicates. Statistic significance is indicated (*, *p* < 0.05; **, *p* < 0.005).

To evaluate the contribution of the host genome to the carrying capacity for foreign DNA, three plasmids of different sizes (S, M and L) were constructed, based on the sequences of pUC19 and pSC101 (Figure 1A). All three plasmids possessed a common replication site (*ori*), a pMB1-derived origin from pUC19, and primarily followed identical replication mechanisms, in a temperature-inducible manner [22]. These plasmids were subsequently transformed into two closely related host strains of different genome sizes, the wild type *E. coli* strain MG1655 and its derivate MDS42, which possesses a reduced genome (*i.e.*, a so-called ‘clean genome’). Exponentially growing host cells bearing these plasmids were repeatedly analyzed at various temperatures (Additional file 1: Figure S1), to alter the plasmid copy numbers. Host cells that lacked plasmids were used as controls for data normalization at each temperature (Additional file 1: Figure S2).

Decreases in growth rate were detected in both host strains when they contained plasmids (Figure 1B, Additional file 1: Figure S2). A trend toward a decrease in host cell growth rate with an increase in plasmid size was observed. The decrease in growth rate caused by the presence of foreign DNA was more significant when the host cells were grown at normal and higher than normal temperatures but was insignificant at the lowest temperature. The results strongly suggest that the burden of foreign DNA was roughly equivalent for the two hosts, although the genome of MDS42 was shorter in length. The costs associated with the replication and/or expression of exogenous DNA were similar for the wild-type and the clean genomes. In addition, the results suggested not only the plasmid copy number (as previously reported [3,4]) but also the plasmid size (Figure 1B) played a role in host growth. The total DNA amount, comprising the information of both copy number and plasmid size, might be a proper parameter to evaluate the host-plasmid relations in common.

### The decrease in growth rate is dependent on the total amount of exogenous DNA

**Figure 2:**
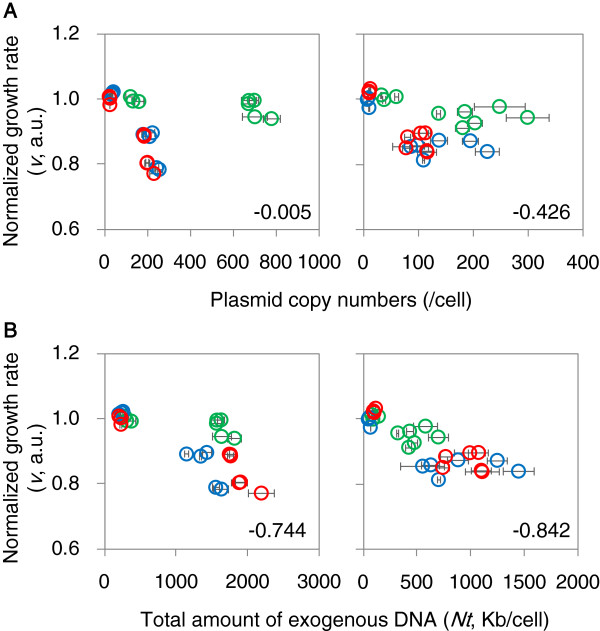
**Relationship between cell growth and the amount of exogenous DNA. A**. Relationship between cell growth and the plasmid copy number. **B**. Relationship between cell growth and the amount of exogenous DNA. The growth rate was normalized to a linear scale. The standard errors and correlation coefficients are given. The left and right panels show the results for strains MG1655 and MDS42, respectively. The color scheme is as described in Figure 1.

In exponentially growing cells, the growth rates of the host cells depended more on the total number of nucleotides contained within the plasmids than on the number of plasmid molecules. Individual analyses of each plasmid-host construct revealed a highly significant negative correlation between normalized growth rates and plasmid copy numbers (*PCNs*) (Additional file 1: Figure S3). Negative correlations were detected for all three plasmids and for both strains. This finding agrees well with the results of previous studies that examined the costs of plasmids to their hosts [9,10]. Intriguingly, even stronger correlations were found between host growth rates and the total length of exogenous DNA (*Nt*) when all three plasmid-host interactions were plotted together (Figure 2), indicating that the cost of plasmid replication for host growth is considerable under the experimental conditions examined. The negative correlation between growth and *PCN* found when combining the data for all three plasmids was significant for MDS42 but not for MG1655 (Figure 2A). The higher significance and greater strength of the negative correlation for *Nt*, compared to *PCN*, was a finding common to both genomes. Both the wild-type and the clean genomes appear to be similarly affected by the burden of foreign DNA.

### Enhanced predictability of the clean genome

**Figure 3:**
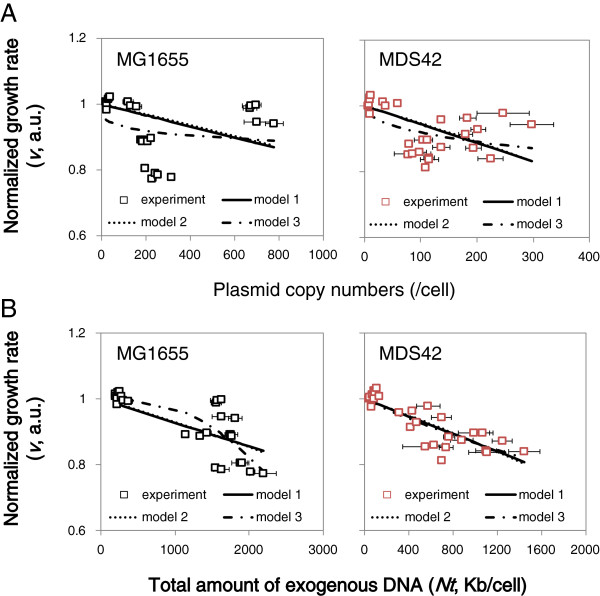
**Comparison among three models. A**. Relationships between cell growth rate and either plasmid copy number. **B**. Relationships between cell growth rate and the total amount of exogenous DNA.. The black and red open squares represent the data for the host strains MG1655 and MDS42, respectively. The bars represent the standard errors of triplicates. The solid, broken and chained lines denote the regression lines for the three models represented by Eqs. 1 (model 1), 2 (model 2) and 3 (model 3), respectively.

To achieve a quantitative understanding of the negative correlations described above, the adherence of the data to a simple linear regression model (model 1) and two models presented in previous studies [23,24] (models 2 and 3) was assessed. The experimental data sets were used to fit these three models individually (Figure 3), and the parameter constants for these models were calculated by applying least squares regression (Table 1). The significances of the models were evaluated by their residual sum of squares (RSS) (Table 2). When considering the relationship between host cell growth and *Nt,* we found that none of the three models provided a better fit to the data than the other models. However, model 3 produced the lowest RSS value when the effect of *PCN* on growth rate was considered. Additionally, for all three models, the analyses of the effect of *Nt* produced lower RSS values compared to those of *PCN*, regardless of the host strain. These theoretical analyses support our new findings (Figure 2) and show that a better quantitative evaluation of the cost of plasmids on host growth is achieved by using the relationship between host growth and the total amount of DNA (*Nt*), as opposed to the plasmid copy number (*PCN*).

**Table 1:** Estimated parameter constants in the models

**Host strain**	***x***	***a***	***c***	***n***	***K***
MG1655	*PCN*	-1.7e-4	3272	0.25	44
MDS42	*PCN*	-5.6e-4	973	0.48	106
MG1655	*Nt*	-7.2e-5	7340	2.87	1.3e10
MDS42	*Nt*	-1.3e-4	4065	0.97	5223

The parameter constants (*a*, *c*, *n* and *K*) for the three models examined (Eqs. 1, 2 and 3, details in the Methods section) were calculated using least squares regression**.***PCN* and *Nt* are the copy number of plasmids and the total amount of plasmid DNA (the number of nucleotides), respectively.

**Table 2:** Residual sums of squares (RSS)

**Host strains**	**Models**	**RSS_** ***PCN***	**RSS_** ***Nt***
MG1655	Eq. 1	0.26	0.09
MG1655	Eq. 2	0.26	0.09
MG1655	Eq. 3	0.18	0.08
MDS42	Eq. 1	0.11	0.04
MDS42	Eq. 2	0.12	0.04
MDS42	Eq. 3	0.09	0.04

The RSS values for the regression analyses were used to evaluate the three models.

Interestingly, the clean genome exhibited better fitting irrespective of the regression model employed. The data for the clean genome fit the models better than the native host genome, and the RSS values were lower for MDS42 than MG1655 for both the *PCN* and *Nt* data (Table 2). Both the increased significance of the correlation between host growth and the amount of exogenous DNA (Figure 3, Table 2) and the higher correlation coefficients (Figure 2) indicate the highly precise control of either cell growth or DNA replication in MDS42. More predictable levels of performance may occur in hosts with fewer nonessential sequences in its genome (*i.e.*, MDS42). The results from this study were consistent with previous findings that have indicated a highly regular, predictable and well-organized behavior for MDS42 at the genome-wide expression level [18,25,26].

### Reduced carrying capacity of the clean genome

Because the linear regression model (model 1) examines the relationship between cell growth rate and the amount of exogenous DNA, the slope (*a*) represents the magnitude of the growth cost of exogenous DNA to the host. A greater decrease in growth was clearly observed in MDS42 compared to MG1655 (Table 1), indicating that the difference in the carrying capacity for exogenous DNA depended on the host genome size. For example, when the linear regression was normalized by the genome size (Additional file 1: Figure S5), in the case of a 50% decrease in fitness, MG1655 could bear an amount of exogenous DNA that was 1.5-fold greater than its genome size, whereas MDS42 could only bear an amount of exogenous DNAs that was equivalent to its genome size. The clean genome might save cellular energy and/or resources for its own genome and restrict energy and/or resources spent on foreign DNA.

**Figure 4:**
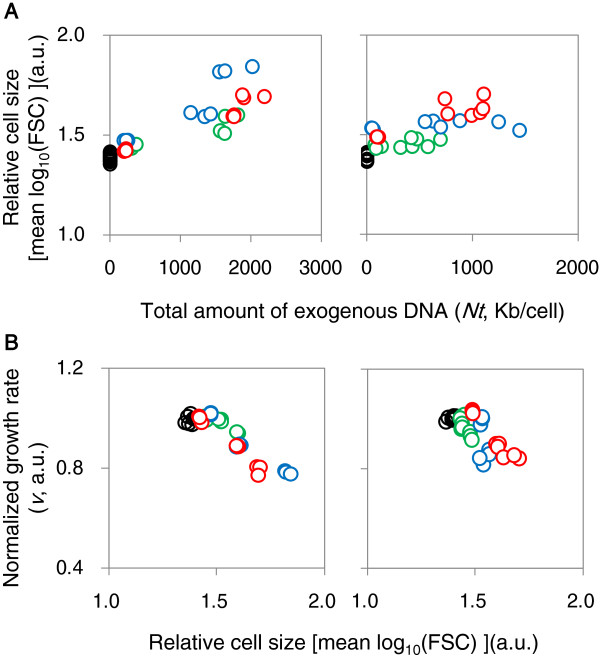
**Relationships between cell size and cell growth and between cell size and the amount of exogenous DNA. A**. Relationship between cell size and the amount of exogenous DNA. **B**. Relationship between cell size and cell growth. Cell size is presented as the mean value of FSC signals measured using flow cytometry. The left and right panels indicate the strains MG1655 and MDS42, respectively. The color scheme is as described in Figure 1.

A similar limit to the carrying capacity of foreign DNA was also detected based on cell size, as estimated by flow cytometry. The relative size of the host cells was positively correlated with the total amount of exogenous DNA (Figure 4A) and negatively correlated with the growth rate (Figure 4B), independent of the host strain. That is, the cell sizes of both host strains increased when they contained foreign DNA. However, MG1655 was able to provide a larger amount of cellular space than MDS42 (Figure 4, Additional file 1: Figure S4), which could directly influence the carrying capacity of foreign DNA. The wild-type genome seemed to be more receptive of foreign DNA, while the clean genome appeared to provide more authoritarian control over the cellular conditions for foreign DNA.

### Restricted replication allows for higher expression levels in the clean genome

**Figure 5:**
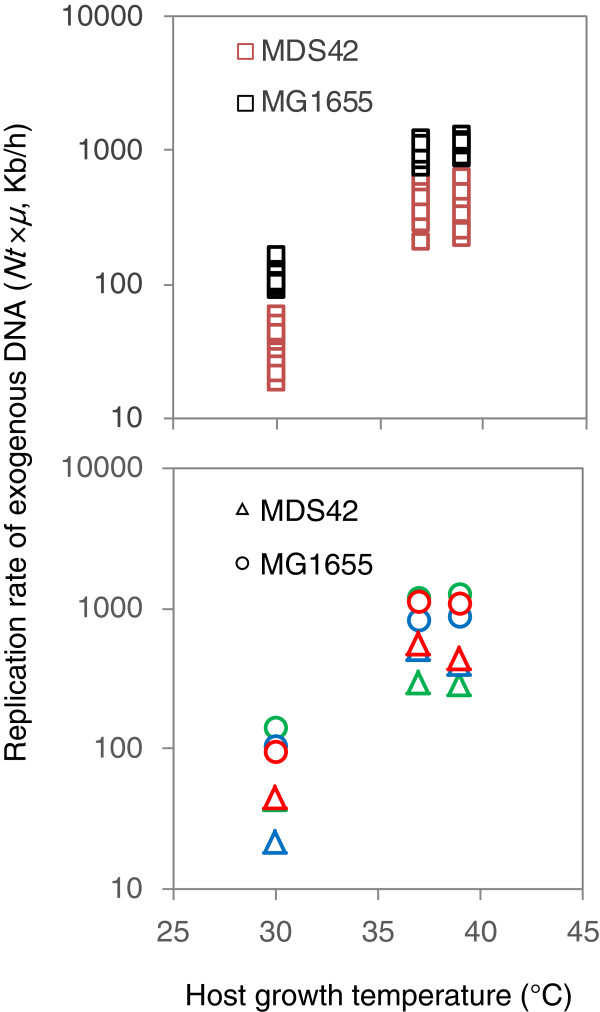
**Comparison of replication rates of exogenous DNA in MG1655 and MDS42.** The replication rates of exogenous DNA in MG1655 (black squares) and MDS42 (red squares) at various growth temperatures (30, 37 and 39°C) are shown in the upper panel. The averaged replication rates for three different sizes of plasmids (S, M and L displayed in green, blue and red, respectively) are shown in the bottom panel. The replication rates of the plasmids in the strains MDS42 and MG1655 are represented by circles and triangles, respectively. The color scheme is as described in Figure 1.

Further analysis demonstrated that the greater decrease in growth with increasing plasmid load (*i.e.*, *PCN* and/or *Nt*) in MDS42 compared to MG1655 was due to a slower replication rate of the plasmids (Figure 5). The total amount of exogenous DNA is determined by the equilibrium between production and dilution. As both strains exhibited approximately equal growth rates under identical conditions (Figure 1B), the dilution rates in the two strains were equivalent. Therefore, the accelerated decreases in the amount of exogenous DNA in MDS42 (Table 1) were caused by decreases in the production (replication rates) of the plasmids. A reduced replication rate of exogenous DNA in MDS42 was observed independent of both the growth temperature (Figure 5, upper) and the sizes of plasmids (Figure 5, bottom). The reduction in host genome size decreased the replication capacity for foreign DNA. This result indicates the possibility that the removal of genes from MG1655 influenced the replication of foreign DNA in this strain. As the previously reported genes specifically related to the DNA and protein productivity [21] were not comprised in the deletions, there might be some novel functions interfering with replication or expression among those deleted genes. In another word, the regions that were removed most likely had a buffering effect, releasing the stress caused by the replication of exogenous DNA and helping to maintain cellular homeostasis. Further systematic tests on different types of genomes along with transcriptome analysis are required to acquire a solid conclusion in mechanisms.

**Figure 6:**
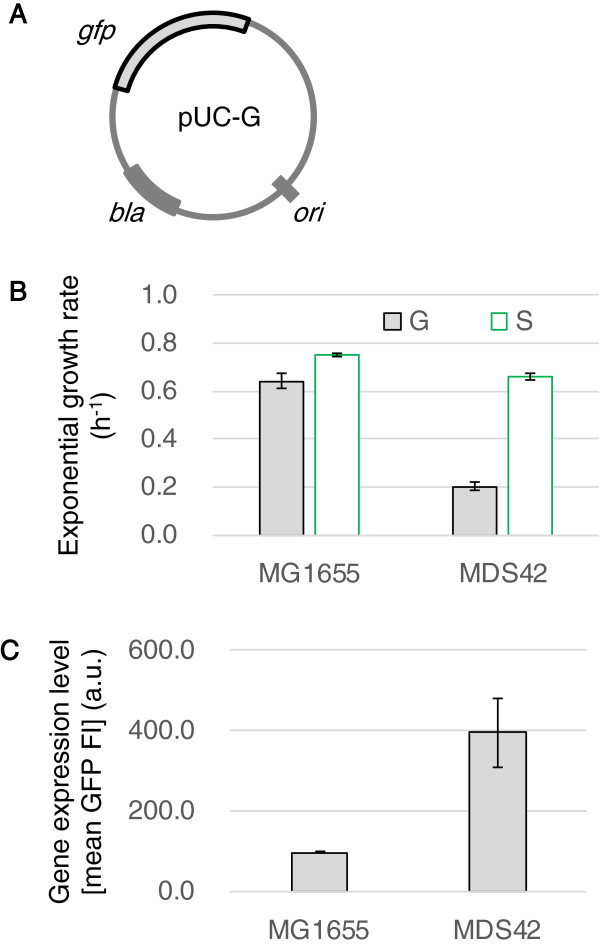
**Cost of gene expression of foreign DNA. A**. Schematic drawing of the plasmid pUC-G. **B**. Exponential growth rates of host cells that contain plasmids. G and S indicate the host cells (MG1655 and MDS42) that contain pUC-G or pUC-S, respectively. **C**. Gene expression levels in MG1655 and MDS42. The mean values of GFP fluorescence intensities (GFP FIs) were calculated to determine the relative levels of gene expression in the host cells, MG1655 and MDS42. The bars represent the standard errors of triplicates. Both increased expression and decreased host growth occurred in MDS42 were highly significant (*p* < 0.001).

In addition, the expression of the foreign DNA could affect the growth burden to a similar extent as replication. To examine the degree to which the presence of a redundant gene decreases growth rates, the plasmid pUC-G (Figure 6A), which is approximately identical in size to pUC-S but carries a nonessential excess gene (*gfp*), was inserted into the host cells. For both strains, the growth rates of the host cells that contained pUC-G were much slower than those of host cells that contained pUC-S (Figure 6B). This result verified that the gene expression of plasmids increased the growth burden beyond the cost paid for the replication of the foreign DNA alone. Importantly, MDS42 was more sensitive to this increase in gene expression; the growth rate of MDS42 was approximately one-third of the growth rate of MG1655 in this experiment. The high expression level of the foreign DNA caused the significant decline in the growth rate of MDS42. An approximately three-fold increase in the GFP expression level was detected in MDS42 (Figure 6C), which was consistent with the decrease in growth rate.

Taken together, the plasmid carrying capacity was lower in MDS42, independent of the growth temperature or the plasmid size (Figure 5), whereas gene expression was definitely higher in MDS42 (Figure 6C), a finding in agreement with previous reports [15,18]. Compared to the wild-type genome, the clean genome assigned a higher priority to the gene expression of foreign DNA than to its replication. Such trade-off like phenomena of increased protein production with decreased plasmid carrying capacity in the reduced genome could be also detected when using the rich medium, despite of the highly accelerated cell growth (Additional file 1: Figure S6). This finding indicates that removing the redundant genes from a host genome can save energy and resources for their direction toward both host genome expression [18] and the expression of foreign genes (Figure 6C), but not DNA replication.

## Conclusions

In summary, the burden of foreign DNA was evaluated in two *E. coli* strains with different genome sizes. The growth rates of both host strains were more negatively correlated with the total amount of exogenous DNA than the plasmid copy numbers. The growth decrease, which was mediated by plasmid replication, was more significant and more theoretically predictable in the strain with the clean genome (MDS42) than that with the wild-type genome (MG1655). The greater decrease in growth that occurred in MDS42 was caused by a slower replication rate of foreign DNA. The results indicate that the MG1655 strain can bear more exogenous DNA but experiences an equivalent loss in growth fitness, whereas MDS42 can restrict the amplification of foreign DNA to maintain its level of growth fitness. Plasmids that are inhibited by constraints in the cellular conditions for replication might benefit from the highly induced level of gene expression found in MDS42. These results indicate that the wild-type strain MG1655 is more suitable for an endosymbiotic application and possesses an advantage in terms of adaptation and evolution, whereas the engineered, clean-genome strain MDS42 more closely resembles a type of living machinery (a controllable cell) and is more suitable for the precise control and production of synthetic materials.

## Methods

### Plasmid construction

The *lacZ* region of pUC19 was removed using the In-Fusion HD Cloning Kit (Clontech) and the *pUC19_del_lacZ_Fw* and *pUC19_del_lacZ_Rv* primers. Both colony PCR and blue-white selection were performed to verify the deletion. The resultant plasmid had a length of 2,334 bp and was named pUC-S. A DNA sequence (4,092 bp) of pSC101 was amplified with the *pSC101_seq_Fw* and *pSC101_seq_Rv* primers. Using the In-Fusion HD Cloning Kit, the resultant fragment was inserted into plasmid pUC19 in the same region where pUC-S was inserted, by using the *pUC19_del_lacZ_linearize_Fw* and *pUC19_del_lacZ_linearize_Rv* primers. This increased the plasmid length of pUC-M to a total length of 6,426 bp. A longer fragment (6,986 bp) from pSC101 that included the tetracycline-resistance gene (Tc^r^) was amplified using the *pSC101_seq_Fw2* and *pSC101_seq_Rv2* primers and then inserted into pUC19 as described above to create the pUC-preL plasmid. Subsequently, a 198-bp fragment was removed from the translation initiation region for Tc^r^ in pUC-preL using the primers *pSC101_del_TcR_Fw* and *pSC101_del_TcR_Rv*, which formed a 9,573-bp-long plasmid, pUC-L. Thus, all three plasmids that were produced had a common site of initiation for replication derived from pUC19, and carried a single gene for ampicillin resistance.

A GFP sequence that includes the promoter P_tet_ was amplified from the pBRgalKGR plasmid (a pBR322 derivative) [26,27] using the *pBR_gfp_kanR_Fw* and *pBR_gfp_kanR_Rv* primers. The *lacZ* region of pUC19 was also removed using the In-Fusion HD Cloning Kit (Clontech) but with a different primer set (*pUC19_del_lacZ_linearize_Fw* and *pUC19_del_lacZ_linearize_Rv2)*, resulting in a 2,334-bp-long fragment, the same length as pUC-S. The two fragments were ligated using the In-Fusion HD Cloning Kit. The *kan*^*R*^ gene sequence (2,866 bp) was removed using the *pUC_del_kanR_Fw* and *pUC_del_kanR_Rv* primers, which finally created the pUC-G plasmid. ECOS™ Competent *E. coli* DH5α cells (Nippon Gene) were used for the cloning and amplification of the plasmids. All the primers used in this study are listed in Additional file 1: Table S1.

### Cell culture and FCM analysis

Cells were cultured in minimal media as described previously [26]. *E. coli* cells that carried the desired plasmids were grown in media that contained an additional 50 μg/mL of ampicillin. The cell concentrations were determined using a flow cytometer (FACSCanto™II, Becton, Dickson and Company) equipped with a 488-nm argon laser and a 515–545-nm emission filter (GFP), as previously reported [26]. The following PMT voltage settings were applied: forward scattering (FSC), 420; side scattering (SSC), 400; and FITC (GFP), 375. The flow cytometry datasets for FSC and GFP, which measure relative cell sizes and gene expression levels, respectively, were analyzed using custom-made R scripts, as described previously [28]. Cell growth was measured during the exponential growth phase; the initial and final cell concentrations were approximately 1-2 × 10^6^ cells/mL and 0.5-2 × 10^8^ cells/mL, respectively, during this stage of growth. The growth rate (*μ*) was calculated according from the initial and final cell concentrations and the length of time between initial and final measurements (8 hours) [18]. The growth data was normalized (*ν*) according to the following equations, as described previously [7,29]:

$$\mu =\ln\left(C_{t}/C_{0}\right)/t$$

$$\nu =\exp\left(\mu_{p}-\mu_{g}\right)$$

### Plasmid copy number and total amount of exogenous DNA

Plasmid copy numbers were determined by qPCR using the StepOnePlus™ Real-Time PCR System (Applied Biosystems) and SYBR Premix Ex Taq™ II (Tli RNaseH Plus) (TaKaRa). The primers (Additional file 1: Table S1) used to amplify the host genome (*dxs_Fw* and *dxs_Rv*) and the plasmid DNA (*bla_Fw* and *bla*_*Rv*) were identical to those used in a previous report [30]. Real-time qPCR reactions were performed using the following program: incubation at 95°C for 30 s, followed by 40 cycles of 95°C for 5 s and 60°C for 60 s. After the final cycle, a melting curve analysis with a temperature gradient of 0.5°C/s from 60°C to 95°C was performed. Aliquots of purified genomic DNA and plasmid DNA were used as the standards for the genome-specific and plasmid-specific qPCR, respectively. The plasmid DNA and genomic DNA were purified using the QIAprep Spin Miniprep Kit (QIAGEN) and the Wizard Genomic DNA Purification Kit (Promega), respectively, according to the manufacturer’s instructions. The abundances of both plasmid DNA and genomic DNA in the *E. coli* cells were determined using standard curves based on the *bla* and *dxs* genes, respectively. The plasmid copy number (*PCN*) and the total amount of exogenous DNA (*Nt*) were calculated according to the following equations:

$$\begin{array}{ll} \mathrm{PCN} & =\mathrm{the}\;\mathrm{abundance}\;\mathrm{of}\;\mathrm{plasmid}\;\mathrm{DNA}/\\ & \;\mathrm{the}\;\mathrm{abundance}\;\mathrm{of}\;\mathrm{genomic}\;\mathrm{DNA} \end{array}$$

Nt=PCN×thelengthofplasmidDNA

### Evaluation of various models

A simple linear regression model was applied to the data for both strains, based on the following equation (Eq. 1, assigned to model 1):

(1) $$f\left(x\right)=\mathrm{ax}+b$$

Here, *a* represents the cost bias of plasmid replication and *b* is the growth rate of the native host cells without plasmids. For example, when the host does not contain a plasmid (*x* = 0), the growth rate is equal to *b* (*i.e.*, *b* = 1). In addition to the simple linear model (Eq. 1), the model primarily used in this study, two other models (Eqs. 2 and 3) have also been previously used [23,24] to estimate the relationship between plasmid copy number (*x*) and host growth rate (*y*):

(2) $$y={\left(1-\frac{x}{c}\right)}^{m}$$

(3) $$y=\frac{K}{K+x^{n}}$$

In all three models (equivalent to Eqs. 1, 2 and 3), *y* is the normalized growth rate and *x* indicates either the copy number of plasmids or the total amount of plasmid DNA. The parameter *c* represents the maximum possible number of plasmids per cell, and the exponent *m* was set to 0.5 according to the recommendation of a previous study [23].

## Competing interests

The authors declare that they have no competing interest.

## Authors’ contributions

YA and BWY conceived the research. YA performed experiments. YA, BWY and ST analyzed the data. BWY wrote the paper. TY provided the experimental and analytical tools and revised the manuscript. All authors approved the final manuscript.

## Supplementary Material

Additional file 1 **Supplementary information containing Figures S1-S6 and Table S1.** Click here for file
