# Supplementary material for: A reduced genome decreases the host carrying capacity for foreign DNA
Source: Microb Cell Fact. 2014 Apr 1;13:49. doi: 10.1186/1475-2859-13-49 (PMC4021254; doi:10.1186/1475-2859-13-49)
Supplement: Additional file 1 — Supplementary information containing Figures S1-S6 and Table S1. [file 1475-2859-13-49-S1.pdf]

A reduced genome decreases the host carrying capacity for foreign DNA

Yuya Akeno *et al.*

Supporting Information

I. Supplementary Figures and Figure Legends (Figures S1–S6) **p. 2-7**

II. Supplementary Table S1 **p. 8**

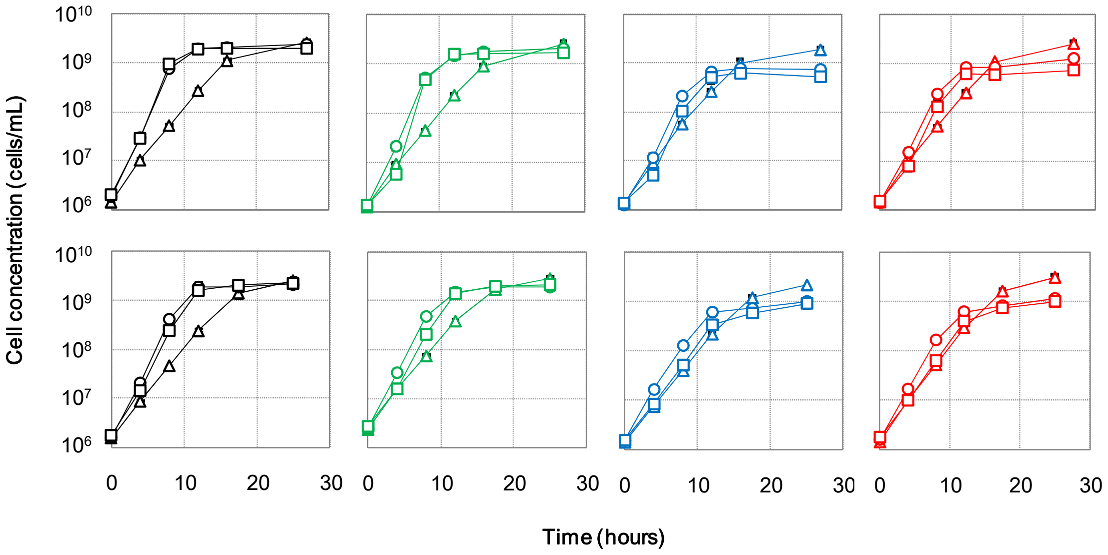

Figure S1. Growth curves. Cells in the exponential phase of growth were transferred to fresh media at an initial concentration of approximately 10^6 cells/mL. The cell concentrations were monitored every four hours. The upper and lower panels show the growth curves for the *E. coli* strains MG1655 and MDS42, respectively. The black, green, blue and red colors indicate cells that either lack plasmids, or contain S plasmids, M plasmids or L plasmids, respectively.

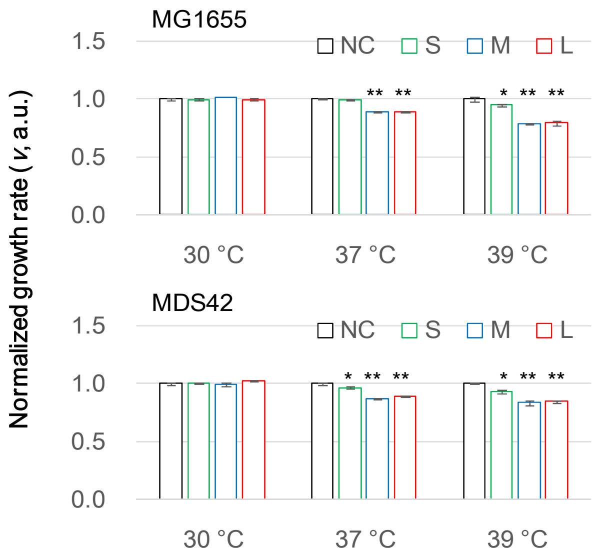

Figure S2. Normalized growth rate. Exponential growth rates evaluated using a logarithmic scale were compared to the growth rates of cells that lacked plasmids and transformed into a linear scale. The color scheme is as described in Figure S1. The bars represent the standard errors of triplicates. Statistic significance is indicated (*, p<0.05; **, p<0.005).

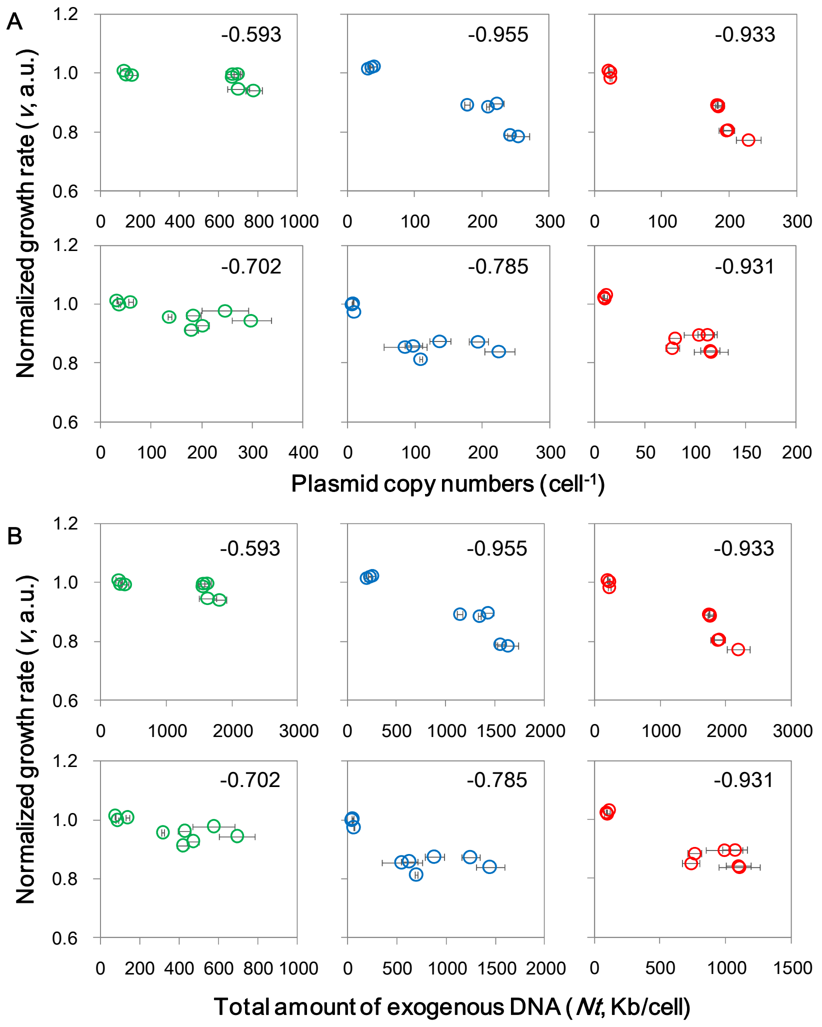

Figure S3. Relationship between exogenous DNAs and host growth. The relationships between growth rates and the plasmid copy number (**A**) or total amount of exogenous DNA (**B**) are plotted separately. The color scheme is as described in Figure S1. The combined plots are shown in Figure 2. The bars represent the standard errors of triplicates.

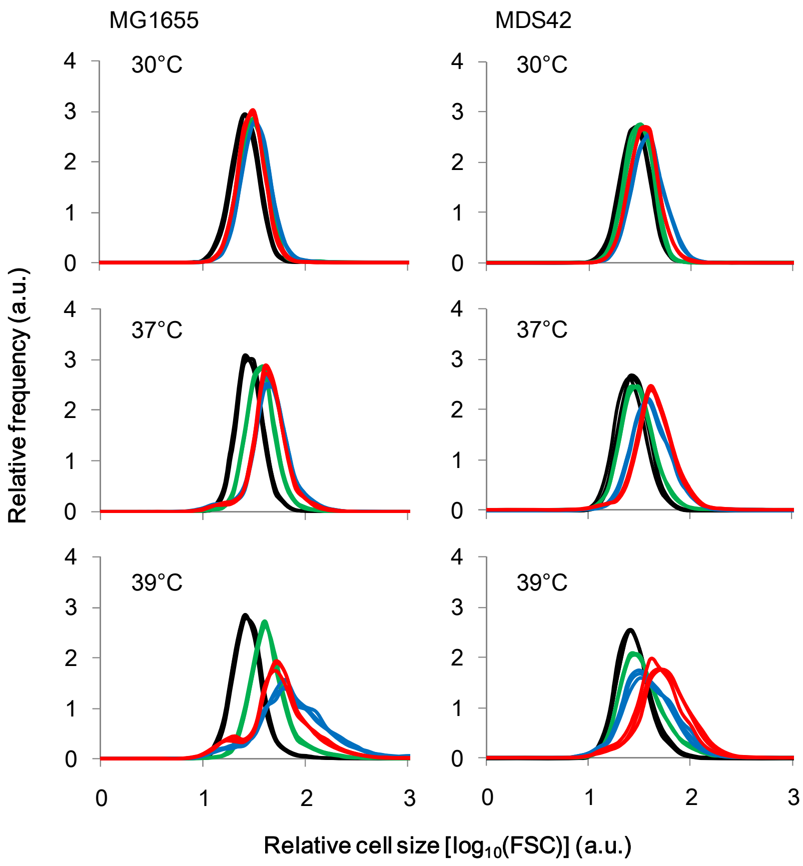

Figure S4. Distributions of relative cell size. Host cells of the MG1655 strain (left panels) and the MDS42 strain (right panels) that were in the exponential phase of growth were measured by flow cytometry. The logarithmic values of FSC were used to compare the relative cell sizes. The color scheme is as described in Figure S1.

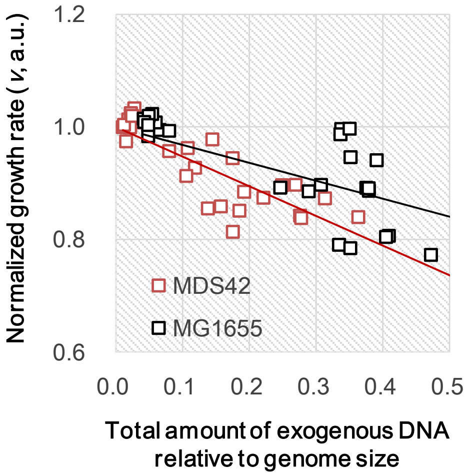

Figure S5. Negative correlation between cell growth and the replication capacity of foreign DNA. The total amount of exogenous DNA was divided by the genome size and plotted against the normalized growth rate. The red and black open squares indicate the MDS42 and MG1655 strains, respectively. The linear regressions are based on Eq. 1 (where b = 1), and the red and black solid lines represent the regression lines for MDS42 and MG1655, respectively.

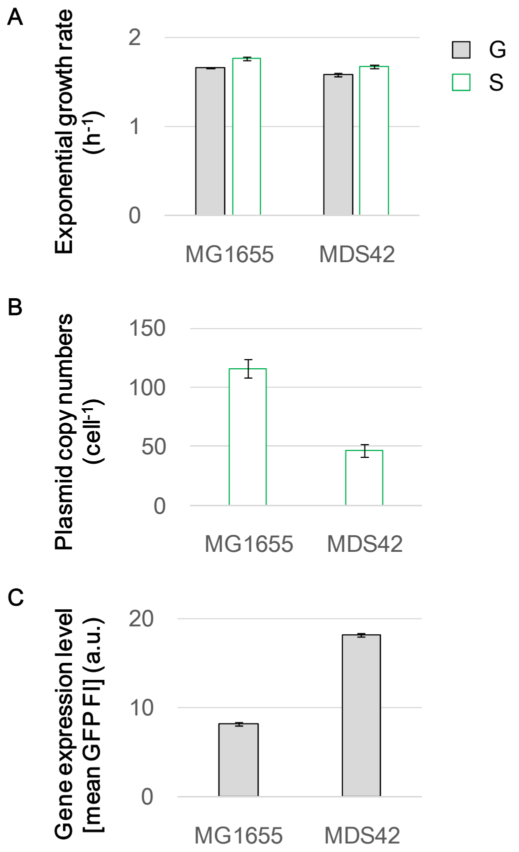

Figure S6. DNA replication and gene expression of foreign DNA in the rich medium. A. Exponential growth rates of host cells that contain plasmids. **B.** Plasmid copy numbers in TB medium. **C.** Gene expression levels in TB medium. The mean values of GFP fluorescence intensities (GFP FIs) were calculated to determine the relative levels of gene expression in the host cells, MG1655 and MDS42. G and S indicate the host cells (MG1655 and MDS42) that contain pUC-G or pUC-S, respectively. The bars represent the standard errors of triplicates. Both increased expression (C) and decreased copy number (B) in MDS42 were highly significant (p<0.001).

Table S1. Primers used in this study. The names and sequences of the primers used for the construction of plasmids and qPCR are listed below.

**Primer name**	**Sequence**
pUC19_del_lacZ_linearize_Fw	TGTCGTGCCAGCTGCATTA
pUC19_del_lacZ_linearize_Rv	AACAGTTGCGCAGCCTGA
pUC19_del_lacZ_linearize_Rv2	GCTGCATGTGTCAGAGGTTT
pSC101_seq_Fw	GGCTGCGCAACTGTTGACAGCATCGCCAGTCACTA
pSC101_seq_Rv	GCAGCTGGCACGACATGCTCATTCGGCGACTTTAAG
pUC19_del_lacZ_Fw	CGCAACTGTTTGTCGTGCCAGCTGCATTAA
pUC19_del_lacZ_Rv	TGGCACGACAAACAGTTGCGCAGCCTGAAT
pSC101_seq_Fw2	GGCTGCGCAACTGTTAATCAAAGCTGCCGACAACA
pSC101_seq_Rv2	GCAGCTGGCACGACAGGGTAAATGGCACTACAGGC
pSC101_del_TcR_Fw	GCAGTTCGAGCCGACAGCATCGCCAGTCACTA
pSC101_del_TcR_Rv	GATGCTGTCGGCTCGAACTGCTTAACGACG
bla_Fw	CTACGATACGGGAGGGCTTA
bla_Rv	ATAAATCTGGAGCCGGTGAG
dxs_Fw	CGAGAAACTGGCGATCCTTA
dxs_Rv	CTTCATCAAGCGGTTTCACA
pBR_gfp_kanR_Fw	TCTGACACATGCAGCGGAAGAGCATGGCCAGAT
pBR_gfp_kanR_Rv	GCAGCTGGCACGACACGGATCAAGCTCTGTAGG
pUC_del_kanR_Fw	AGCTCACTCAAAGGCGGTAA
pUC_del_kanR_Rv	ACTTCGAACTGCAGGTCGTC
